# Correction: Association of race and health insurance in treatment disparities of colon cancer: A retrospective analysis utilizing a national population database in the United States

**DOI:** 10.1371/journal.pmed.1003937

**Published:** 2022-02-23

**Authors:** Scarlett Hao, Rebecca A. Snyder, William Irish, Alexander A. Parikh

In the title of this article, the word “population” should be “hospital-based.” The correct title is: Association of race and health insurance in treatment disparities of colon cancer: A retrospective analysis utilizing a national hospital-based database in the United States. The correct citation is: Hao S, Snyder RA, Irish W, Parikh AA (2021) Association of race and health insurance in treatment disparities of colon cancer: A retrospective analysis utilizing a national hospital-based database in the United States. PLoS Med 18(10): e1003842. https://doi.org/10.1371/journal.pmed.1003842
