# Supplementary material for: Association of race and health insurance in treatment disparities of colon cancer: A retrospective analysis utilizing a national population database in the United States
Source: PLoS Med. 2021 Oct 25;18(10):e1003842. doi: 10.1371/journal.pmed.1003842 (PMC8575307; doi:10.1371/journal.pmed.1003842)
Supplement: S1 Table — (DOCX) [file pmed.1003842.s001.docx]

**NCDB – Interaction Race and Health Insurance – Colon Cancer**

**Primary Aim**: Is the impact of health insurance different based on race for the treatment of colon cancer?

**Study population**:

1. Primary study population: AJCC Stage I-III colon cancer patients within NCDB from 2006-2016.
2. Secondary study population: AJCC stage III colon cancer that received surgical resection within NCDB from 2006-2016 (subgroup to study adjuvant chemotherapy)

**Identification of study population**:

- - Sequence number 00 (patient’s 1^st^ cancer)
  - Stage I-III (ANALYTIC_STAGE_GROUP value = 1-3)
  - Insurance status (INSURANCE_STATUS value = 0-4)
- Race/Ethnicity (RACE value=1 (white), 2 (black), 3-99 (other) combine with SPANISH_HISPANIC_ORIGIN value = 0 for Non-Hispanic, 1-8 for Hispanic) = NHW, NHB, Hispanic, Non-Hispanic Other
  - *Secondary population: stage III (ANALYTIC_STAGE_GROUP value = 3), Received resection (RX_SUMM_SURG_PRIM_SITE value = 20-90)
  - Histology of adenocarcinoma ONLY

**Primary outcomes**:

1. Receipt of surgery (RX_SUMM_SURG_PRIM_SITE value = 20-90)
2. Receipt of adjuvant chemotherapy (secondary study population only) (RX_SUMM_SYSTEMIC_SUR_SEQ value =3, RX_SUMM_CHEMO value = 01,02,03)

**Analysis**:

1. MVA of factors associated with receipt of surgery
   1. Interaction of race/health insurance
   2. Stratify by race/health insurance
2. MVA of factors associated with receipt of adjuvant chemotherapy in surgical resected patients
   1. Interaction of race/health insurance
   2. Stratify by race/

**Primary variables of interest**:

Primary Payer

Race/Ethnicity

**Confounders**:

- Facility type
- Age
- Sex
- Income
- Education
- Urban/rural
- Travel distance (great circle distance)
- Charlson-Deyo comorbidity score
- Year of diagnosis
- Stage: ANALYTIC_STAGE_GROUP value = 1-3
- Tumor site: PRIMARY_SITE value = C180-189 for colonic origin
- Grade: GRADE value = 1-4
