# Supplementary material for: Association of race and health insurance in treatment disparities of colon cancer: A retrospective analysis utilizing a national population database in the United States
Source: PLoS Med. 2021 Oct 25;18(10):e1003842. doi: 10.1371/journal.pmed.1003842 (PMC8575307; doi:10.1371/journal.pmed.1003842)
Supplement: S2 Table — (DOCX) [file pmed.1003842.s002.docx]

STROBE Statement—Checklist of items that should be included in reports of ***cross-sectional studies***

|  | Item No | Recommendation | Section ; ¶ No |
| --- | --- | --- | --- |
| **Title and abstract** | 1 | (*a*) Indicate the study’s design with a commonly used term in the title or the abstract  Line 46: “in this cross-sectional study” | Abstract; subsection methods |
|  |  | (*b*) Provide in the abstract an informative and balanced summary of what was done and what was found  Lines 46-63: Describes the retrospective cross-sectional study, statistical analysis, and findings | Abstract; subsection methods |
| Introduction | | | |
| Background/rationale | 2 | Explain the scientific background and rationale for the investigation being reported  Lines 69-89: Briefly presents existing literature on both racial disparities and insurance disparities in cancer care. The rationale for the investigation was to evaluate the impact of one factor on the other. | Introduction; ¶1, 2 |
| Objectives | 3 | State specific objectives, including any prespecified hypotheses  Lines 90-92: Primary aim and hypothesis are specified | Introduction; ¶3 |
| Methods | | | |
| Study design | 4 | Present key elements of study design early in the paper  Lines 46-63: Abstract briefly presents the key elements of study design. Methods section in the manuscript separates this into several subsection | Abstract; subsection methods |
| Setting | 5 | Describe the setting, locations, and relevant dates, including periods of recruitment, exposure, follow-up, and data collection  Lines 95-129: Data sources and specific data collected are detailed. | Methods; subsections Data Source, Study Population, Variables and Outcomes |
| Participants | 6 | (*a*) Give the eligibility criteria, and the sources and methods of selection of participants  Lines 106-114: Inclusion criteria are described | Methods; subsection Study Population |
| Variables | 7 | Clearly define all outcomes, exposures, predictors, potential confounders, and effect modifiers. Give diagnostic criteria, if applicable  Lines 116-155: Outcomes and their associated analyses are described. | Methods; subsection Variables and Outcomes, Statistical Analysis |
| Data sources/ measurement | 8* | For each variable of interest, give sources of data and details of methods of assessment (measurement). Describe comparability of assessment methods if there is more than one group  Lines 95-104: the data source is described here. How each variable is provided in the NCDB PUF is publicly available on the NCDB website | Methods; subsections Data Source |
| Bias | 9 | Describe any efforts to address potential sources of bias  Lines 129-142: the multivariable regression was built to address confounding variables | Methods; subsection Statistical Analysis, ¶2 |
| Study size | 10 | Explain how the study size was arrived at  Figure 1 describes the steps of inclusion and exclusion | Figure |
| Quantitative variables | 11 | Explain how quantitative variables were handled in the analyses. If applicable, describe which groupings were chosen and why  Lines 116-155: Specific variables are named and how they are presented is described | Methods; subsection Variables and Outcomes, Statistical Analysis |
| Statistical methods | 12 | (*a*) Describe all statistical methods, including those used to control for confounding  Lines 131-155: Multivariable main model and joint effects model described | Methods; subsection Statistical Analysis |
|  |  | (*b*) Describe any methods used to examine subgroups and interactions  Lines 139-155: Joint effects models described to examine the interaction effect | Methods; subsection Statistical Analysis, ¶2 |
|  |  | (*c*) Explain how missing data were addressed  Lines 134-135: “Missing data was considered as a separate category” | Methods; subsection Statistical Analysis, ¶1 |
|  |  | (*d*) If applicable, describe analytical methods taking account of sampling strategy |  |
|  |  | (*e*) Describe any sensitivity analyses |  |
| Results | | | |
| Participants | 13* | (a) Report numbers of individuals at each stage of study—eg numbers potentially eligible, examined for eligibility, confirmed eligible, included in the study, completing follow-up, and analysed  Figure 1 also includes the # of individuals at each stage of the study population selection | Figure |
|  |  | (b) Give reasons for non-participation at each stage  This is indicated in Figure 1 | Figure |
|  |  | (c) Consider use of a flow diagram  Figure 1 is a flow diagram | Figure |
| Descriptive data | 14* | (a) Give characteristics of study participants (eg demographic, clinical, social) and information on exposures and potential confounders  This information is presented in Table 1 | Table 1 |
|  |  | (b) Indicate number of participants with missing data for each variable of interest  This information is presented where available in Table 1 | Table 1 |
| Outcome data | 15* | Report numbers of outcome events or summary measures  This is presented in Table 2 | Table 2 |
| Main results | 16 | (*a*) Give unadjusted estimates and, if applicable, confounder-adjusted estimates and their precision (eg, 95% confidence interval). Make clear which confounders were adjusted for and why they were included  Unadjusted outcome data with precision statistics are presented in Table 2 | Table 2 |
|  |  | (*b*) Report category boundaries when continuous variables were categorized |  |
|  |  | (*c*) If relevant, consider translating estimates of relative risk into absolute risk for a meaningful time period |  |
| Other analyses | 17 | Report other analyses done—eg analyses of subgroups and interactions, and sensitivity analyses  Adjusted analyses are presented in Figure 2 and Table 3  The joint effects model evaluating interaction effect is presented in Table 4 | Figure, Table 3 and 4 |
| Discussion | | | |
| Key results | 18 | Summarise key results with reference to study objectives  Lines 242-253: discussion summarizes the key results | Discussion, ¶1 |
| Limitations | 19 | Discuss limitations of the study, taking into account sources of potential bias or imprecision. Discuss both direction and magnitude of any potential bias  Lines 303-313: Limitations are presented | Discussion, ¶6 |
| Interpretation | 20 | Give a cautious overall interpretation of results considering objectives, limitations, multiplicity of analyses, results from similar studies, and other relevant evidence  Lines 254-302: Interpretation of results in light of other similar and relevant studies is provided | Discussion, ¶2-5 |
| Generalisability | 21 | Discuss the generalisability (external validity) of the study results  Lines 312-313: Generalizability is limited by the facilities that report to the NCDB | Discussion, ¶6 |
| Other information | | | |
| Funding | 22 | Give the source of funding and the role of the funders for the present study and, if applicable, for the original study on which the present article is based  Funding information is provided separately in the submission process | n/a |

*Give information separately for exposed and unexposed groups.

**Note:** An Explanation and Elaboration article discusses each checklist item and gives methodological background and published examples of transparent reporting. The STROBE checklist is best used in conjunction with this article (freely available on the Web sites of PLoS Medicine at http://www.plosmedicine.org/, Annals of Internal Medicine at http://www.annals.org/, and Epidemiology at http://www.epidem.com/). Information on the STROBE Initiative is available at www.strobe-statement.org.
