# Supplementary material for: Association of race and health insurance in treatment disparities of colon cancer: A retrospective analysis utilizing a national population database in the United States
Source: PLoS Med. 2021 Oct 25;18(10):e1003842. doi: 10.1371/journal.pmed.1003842 (PMC8575307; doi:10.1371/journal.pmed.1003842)
Supplement: Attachment — Submitted filename: Response to Reviewers .docx [file pmed.1003842.s003.docx]

**Response to the Editor/Reviewers:**

Editorial comments:
1)      Please revise your title according to PLOS Medicine's style. Your title must be nondeclarative and not a question. It should begin with main concept if possible. "Effect of" or “risk of” should be used only if causality can be inferred, i.e., for an RCT. Please include the setting. In addition, please place the study design ("A retrospective study,") in the subtitle (i.e., after a colon).

**We have revised the title as requested.**

2)      Abstract:
a)      Please ensure that all numbers presented in the abstract are present and identical to numbers presented in the main manuscript text.

**This has been corrected.**

b)      Please include the study setting.

**This has been included.**

c)      Please include the specific important dependent variables that are adjusted for in the analyses.

**This has been added to the Methods and Findings section.**

d)      Please quantify the main results (with 95% CIs and p values).

**Data has been added to the interaction effect results for patients with private and Medicare insurance receiving resection or chemotherapy.**

3)      Did your study have a prospective protocol or analysis plan? Please state this (either way) early in the Methods section.
a)      If a prospective analysis plan (from your funding proposal, IRB or other ethics committee submission, study protocol, or other planning document written before analyzing the data) was used in designing the study, please include the relevant prospectively written document with your revised manuscript as a Supporting Information file to be published alongside your study, and cite it in the Methods section. A legend for this file should be included at the end of your manuscript.

**This has been included in the revised manuscript, legend included at the end**

b)      If no such document exists, please make sure that the Methods section transparently describes when analyses were planned, and when/why any data-driven changes to analyses took place.
c)      In either case, changes in the analysis-- including those made in response to peer review comments-- should be identified as such in the Methods section of the paper, with rationale.
4)      For this observational study, in the manuscript text, please indicate: (1) the analytical methods by which you planned to test your hypothesis, (2) the analyses you actually performed, and (3) when reported analyses differ from those that were planned, transparent explanations for differences that affect the reliability of the study's results. If a reported analysis was performed based on an interesting but unanticipated pattern in the data, please be clear that the analysis was data-driven.

**This has been included in the discussion – specifically the finding that Hispanic patients were less likely to undergo resection, but the odds of receiving adjuvant chemotherapy was similar to NHW. This was different than our finding for NHB patients.**

5)      In statistical methods, please refer to any post-hoc corrections to correct for multiple comparisons during your statistical analyses. If these were not performed please justify the reasons. Please refer to our statistical reporting guidelines for assistance (<https://nam02.safelinks.protection.outlook.com/?url=https%3A%2F%2Fjournals.plos.org%2Fplosone%2Fs%2Fsubmission-guidelines.%23loc-statistical-reporting&amp;data=04%7C01%7Cparikha19%40ecu.edu%7Cdbc8ed4bc8504bc998c308d935baaf5c%7C17143cbb385c4c45a36ac65b72e3eae8%7C0%7C0%7C637599898199549711%7CUnknown%7CTWFpbGZsb3d8eyJWIjoiMC4wLjAwMDAiLCJQIjoiV2luMzIiLCJBTiI6Ik1haWwiLCJXVCI6Mn0%3D%7C1000&amp;sdata=0Khwx2XKhx3Nxtvg4wEeb9SFaLe2z6%2BSCKQbYu3PxOQ%3D&amp;reserved=0>)

**We did not perform a correction for multiple comparisons given that the study is observational and exploratory. To put this into proper statistical context, we have revised the Introduction to explicitly state the study intent.**

6)      Thank you for providing your STROBE checklist. Please replace the page numbers with paragraph numbers per section (e.g. "Methods, paragraph 1"), since the page numbers of the final published paper may be different from the page numbers in the current manuscript.

**This has been adjusted.**

7)      Your study is observational and therefore causality cannot be inferred. Please remove language that implies causality. Instead, refer to associations consistently throughout the text.

**The manuscript has been reviewed and any suggestion of causality has been removed.**

8)      Some reviewer concerns include the lack of individual level SES and some health care variables. These might affect inference and policy recommendations with regards to health system role vs other drivers of disparity. Please consider some sensitivity analyses to address this or at minimum calculate the e-value to determine how large the confounders would have to be to invalidate the race effect.

**The revised Table 4 includes the E-value for the odds ratio using the described in VanderWeele and Ding (2017) [VanderWeele TJ & Ding P (2017). Sensitivity analysis in observational research: introducing the E-value. Annals of Internal Medicine, 167(4), 268-274.] The E-value, as noted by the reviewer is the minimum strength of both the confounder associations that must be present, above and beyond the measured covariates, for an unmeasured confounder to explain away an association. For example, the odds ratio of surgical resection for non-Hispanic Black versus non-Hispanic White who are on Medicare is 0.59 with an E-value 1.92. The observed OR of 0.59, therefore could be explained away by an unmeasured confounder that was associated with both race and surgical resection by an OR of 1.93-fold each, above and beyond the measured confounders, but weaker confounding could not do so. Relatively strong confounding associations would be needed to completely explain away the observed race-surgical resection association of OR=0.59. Unfortunately, it is difficult to know what constitutes weak (or strong) association of unmeasured confounders with race and surgical resection, above and beyond the measured confounders included in the model.**

9)      In your statistical analyses, please use hierarchical/ multilevel models or generalized estimating equations given that nationwide data is likely clustered at state/ county and hospital levels. The potential clustering of data (e.g., among patients from the same locality or hospital) would result in spurious effect estimates and standard errors.

**As suggested by the reviewer, we fit a generalized estimating equations (GEE) binary logit model clustered within facility, assuming an exchangeable working correlation structure. Using the GEE results, we updated Table 3 and Table 4. We have revised the text in the Statistical Analysis section accordingly, describing the GEE procedure.**

10)     Please provide 95% CIs and p values for all estimates in the text and tables.

**This has been included**

11)     Please specify the significance level used (eg, P<0.05, two-sided) and the statistical test used to derive a p value.

**This has been included in the Methods**

12)     Please do not report P<0.01; report as P < 0.001.

**This has been corrected.**

13)     Please define the abbreviations in Tables and Figure e.g., AJCC, NOS, HS,Dx, SD, etc

**Abbreviation explanations have been added to the bottom of each table and in figure captions.**

14)     Please indicate in the figure caption the meaning of the bars and whiskers in Figure 2

**This has been added to the figure caption.**

Comments from the reviewers:

Reviewer #1: This study aims to determine the interaction of race and insurance with CC treatment disparities.

Comments:

The authors have appropriately provided the STROBE checklist in the supplementary material.

"The National Cancer Database (NCDB) sponsored by the American College of Surgeons and American Cancer Society gathers data from more than 1500 Commission on Cancer (CoC)-accredited facilities in the United States."
Did the authors consider accounting for clustering by health care facility within the analysis?

**Please see response above.**

"The primary outcomes of interest were: 1) receipt of surgical resection and 2) receipt 124 of adjuvant chemotherapy in the subgroup of eligible patients with resected stage III CC, 125 stratified by race and insurance."
Can the authors please comment on whether it is possible to attain information on the offer of treatment to patients, and to therefore model patient uptake rates accounting for this?

**Unfortunately, it is not possible to accurately assess reasons for why surgery or chemotherapy were not given as a significant number of patients have missing values for this variable within the NCDB. This has been added as a limitation.**

"Continuous variables are described by the number of non-missing observations, mean, standard deviation, median, and 25th and 75th percentiles. Categorical variables are described overall and by cohort with the number of patients and percentage for each category. Missing data was considered as a separate category".
The authors have used valid statistical descriptors for the data types in hand.

"To adjust for confounding and estimate the association of outcome to covariates, data was fit using multivariable binary logistic regression models. Two models were fit to the data: a main effects model with additive terms for race and insurance status adjusted for additional covariates and a joint effects model with a two way interaction term for race and insurance also adjusted for additional covariates. These included: age, race, sex, insurance status, income level, education, rurality, comorbidity, distance traveled for care, and tumor grade. The joint effects model was used to evaluate the effect of race on outcome within levels of insurance status. Adjusted odds ratios (OR) and 95% confidence intervals (CI) are provided as measures of strength of association and precision, respectively. The joint effect of race and insurance status on outcomes was tested using Wald's Chi-square on 12 degrees of freedom."
With reference to my earlier comment regarding possible clustering in the data by health care facility, did the authors consider a modelling approach (such as multilevel modelling, for example) that takes this into account?

**Please see our response above (Editorial comment 9).**

The authors have done well to include covariates in an attempt to adjust for potential confounding in the models.  Can they further comment on whether a measure of deprivation is available for inclusion?

**We appreciate the reviewer’s comments, unfortunately a measure of deprivation is not available or can accurately be calculated within the current NCDB.**

Tables 1 and 2:  Did the authors consider statistically testing for differences between racial cohorts and insurance groups, and presenting these results within the Tables and in support of statements in the Results text?

**This has been added to the demographic tables 1 and 2.**

Can the authors please comment on and discuss how "When evaluated by race, 67.6% of patients of NHW race received adjuvant chemotherapy compared to 70.9% of patients of NHB race" (Table 2) matches up with the reduced odds seen in Tables 3 and 4?

**The discrepancy between unadjusted data such as in Table 2 and adjusted data such as in Tables 3 and 4 is the fundamental reason for why we utilized a multivariable analysis. Unadjusted data does not consider many influential and confounding factors such as patient comorbidity or ability to afford treatment that could explain differences in outcomes, whereas multivariable regression adjustment attempts to control for as many confounders as are available in the dataset.**

Reviewer #2: Thank you for the opportunity to review this paper about the intersection of race and health insurance in the treatment of colon cancer. This paper makes an important contribution to the literature by examining whether racial disparities persist after stratifying by health insurance coverage. Below are suggestions for improving the paper.
*       Throughout the paper, the authors use the term "non-White." This term centers whiteness and likely categorizes patients in a way they would not self-identify. Furthermore, using this term may imply to some readers that patients of all races other than white were grouped together in analyses, which does not appear to be the case for this study. Please consider being more precise about the racial and ethnic groups of patients (e.g., non-Hispanic Black and Hispanic) when describing study results.

**We greatly appreciate the reviewer's consideration for racial/ethnic identity and have striven to correct all uses of the term "non-White."**

*       In the methods, please clarify the approach used to address missing data.

**Missing/unknown variables were treated as a separate category for demographic purposes as recommended in the STROBE guidelines. The multivariable adjusted analysis excludes the missing/unknown category for all included covariates. This has been added to the Methods section in the Statistical Analysis subsection.**

*       In the limitations, it is also important to note that some variables (e.g., educational attainment) were derived using data from the patient's zip code and may not accurately represent the patient's actual education level.

**This has been added to the limitations paragraph in the Discussion section.**

*       In the discussion, the authors note that, "Third, although the NCDB is based on rigorous comprehensive data collection, the dataset lacks information regarding specific social determinants of health, thereby limiting a more comprehensive analysis of other social factors likely to affect health care access." This is an important point that warrants more discussion in the paper (e.g., in the introduction and conclusion). It may be helpful to provide key examples of prior evidence about social factors and determinants of health (e.g., racism in health care) that likely influence these outcomes. It is important to be specific about these factors so that readers will not make inaccurate assumptions about potential causes of racial disparities in cancer outcomes.

**We strongly agree on the importance of social determinants of health and their impact on cancer care and have added additional content to the introduction and conclusion.**

*       In the conclusion, please clarify what is meant by "inadequate" insurance coverage.

**The wording has been revised to improve clarification.**

*       Please revise the following sentence as there appears to be a typo: Uninsured rates are particularly high among rural residents of non-White racial minority and correlate with high rates of self-reported poor health.

**This has been revised.**

Reviewer #3: Dear authors,
This is a well-written manuscript assessing the associations between race/ethnicity, insurance status and colon cancer outcomes. The study's main findings are than non-Hispanic Black patients are less likely to undergo resection for colon cancer or to receive chemotherapy in comparison to non-Hispanic white patients. The study's strongest strength is the use of a large, nationally-representative dataset. Additionally, the manuscript is well organized and written clearly enough to be accessible to health professionals that do not specialize in colon cancer research. However, the study has a number of weaknesses, including the lack of data on important confounders, such as individual-level SES and information about the patients' health systems. Also, the study contained limited granularity in the number of racial and ethnic groups examined.
Importantly, this manuscript is unlikely to directly or substantially affect public health policy. The racial disparity experienced by non-Hispanic Blacks in health care access and colorectal cancer screening, diagnosis and treatment has been well-documented in the literature. The results of this study do not provide a substantial advance over existing knowledge in colorectal cancer outcomes research.
Specific suggestions for the manuscript are provided below:
1. Abstract - the second sentence refers to "these disparities", however disparities have not yet been defined in the text. Please rephrase to define the disparities of interest for the manuscript.

**The wording has been revised for clarification.**

2. General - please be consistent in the use of colon vs colorectal cancer

**Although the focus of our study was cancer, useveral studies that we reference group colon and rectal cancer in their studies. All of these references have been re-reviewed and have been changed to colon cancer when appropriate.**

3. General - please use ethnicity (not race) when referring to comparisons to Hispanic patients

**Because we have grouped race and ethnicity factors in dividing our cohorts (NHW, NHB, Hispanic),** **"race" has been replaced with "race/ethnicity."**

4. Introduction - please update 1st paragraph with data from 2021, and there are racial groups that have lower CC rates than non-Hispanic whites (e.g. API), please revise.

**The most recent statistics have been added. The umbrella term non-White has been removed and specific mention made of NHB and Hispanic race, recognizing that there are minority races as the reviewer indicates which have lower incidence compared to NHW patients.**

5. Introduction - 2nd paragraph: it is not useful to describe all non-white Americans as a monolith. Please be more specific about the disparities being addressed.

**Instances of the phrase "non-White" have been removed in favor of more specific racial/ethnic groups.**

6. Methods - does the dataset include indication for why patients did not receive surgery/chemotherapy?

**Unfortunately, there is too much missing data within the NCDB in regard to reasons why a patient did not receive a particular treatment.** **This has been added as a limitation.**

7. Methods/Discussion - educational attainment measured at the zip code-level was included as a covariate. Please discuss how this variable may be associated with the outcomes of the study.

**This particular variable has infrequently studied with regards to colon cancer using the NCDB. One study found an association between this variable and lower odds of undergoing surgery at a high-volume hospital for esophageal, liver, and pancreatic cancer, although not seen for colon cancer. It is a socioeconomic variable that is measured by the American Community Survey and included in neighborhood socioeconomic indices which do have an association with colon cancer disparities:**

- **Ellis L, Canchola AJ, Spiegel D, Ladabaum U, Haile R, Gomez SL. Racial and Ethnic Disparities in Cancer Survival: The Contribution of Tumor, Sociodemographic, Institutional, and Neighborhood Characteristics. J Clin Oncol. 2018;36(1):25-33. doi:10.1200/JCO.2017.74.2049**
- **Snyder RA, Hu CY, Zafar SN, Francescatti A, Chang GJ. Racial Disparities in Recurrence and Overall Survival in Patients With Locoregional Colorectal Cancer. J Natl Cancer Inst. 2021;113(6):770-777. doi:10.1093/jnci/djaa182**

**With respect to cancer care overall, health literacy does have an association with reduced screening uptake, delay in diagnosis, lower rates of treatment, with potential consequences on overall survival (Coughlin SS, Vernon M, Hatzigeorgiou C, George V. Health Literacy, Social Determinants of Health, and Disease Prevention and Control. J Environ Health Sci. 2020;6(1):3061). Unfortunately, the percent no high school degree by ZIP code variable is the only surrogate marker available for this type of data. This is reflected in the limitations paragraph.**


Any attachments provided with reviews can be seen via the following link:
