# Supplementary material for: Association of race and health insurance in treatment disparities of colon cancer: A retrospective analysis utilizing a national population database in the United States
Source: PLoS Med. 2021 Oct 25;18(10):e1003842. doi: 10.1371/journal.pmed.1003842 (PMC8575307; doi:10.1371/journal.pmed.1003842)
Supplement: Attachment — Submitted filename: PLOS Med Response to Reviewers 9-22.docx [file pmed.1003842.s004.docx]

Response to Reviewers/Editors:

Requests from Editors:
1)      Please include the study setting/ country in the title.

*This has been added.*

2)      Please include the study country in the abstract’s “Method and Findings” section.

*This has been added (line 45)*

3)      Please integrate the author summary with the main text. This should follow the abstract.

*This has been added (lines 67-100)*

4)      In the main text and tables, please provide both Odds Ratios (OR) and adjusted ORs for unadjusted and adjusted analyses.

*This has been added.*

5)      Line #161 “… some…” instead of “…som…”

*This has been corrected.*

6)      References - Please ensure that journal name abbreviations consistently match those found in the National Center for Biotechnology Information (NCBI) databases, and are appropriately formatted and capitalized.

*All references have been checked and corrected.*

Comments from Reviewers:

Reviewer #1: The authors have satisfactorily responded to each comment in turn, amending the analytical approach and presenting statistical tests accordingly.

Reviewer #2: Thank you for the opportunity to review the revised version of this manuscript. The authors have addressed my previous concerns and suggestions. This paper will make a useful contribution to the field, especially now that the authors revised their analytic approach and clarified a number of points throughout the paper.

*We are grateful to Reviewers #1 and #2 for their critical appraisal of our work.*
